# Supplementary material for: Logical design of oral glucose ingestion pattern minimizing blood glucose in humans
Source: NPJ Syst Biol Appl. 2019 Sep 2;5:31. doi: 10.1038/s41540-019-0108-1 (PMC6718521; doi:10.1038/s41540-019-0108-1)
Supplement: Supplementary file 1 — Spplemental Figures and Tables [file 41540_2019_108_MOESM1_ESM.pdf]

Supplementary Information for "Logical design of oral glucose ingestion pattern minimizing blood glucose in humans"

Masashi Fujii[1,2,3]†,, Yohei Murakami[4]†, Yasuaki Karasawa[5,6]†, Yohei Sumitomo[2], Suguru Fujita[2], Masanori Koyama[7], Shinsuke Uda[8], Hiroyuki Kubota[8], Hiroshi Inoue[9], Katsumi Konishi[10], Shigeyuki Oba[4], Shin Ishii[4,11], Shinya Kuroda[1,2,11]*

[1]Molecular Genetic Research Laboratory, Graduate School of Science, The University of Tokyo, Tokyo, 113-0033, Japan.
[2]Department of Biological Sciences, Graduate School of Science, The University of Tokyo, Tokyo, 113-0033, Japan.
[3]Present address: Department of Integrated Sciences for Life, Graduate School of Integrated Sciences for Life, Hiroshima University, Hiroshima, 739-8526, Japan.
[4]Department of Systems Science, Graduate School of Informatics, Kyoto University, Kyoto, 606-8501, Japan.
[5]Department of Neurosurgery, The University of Tokyo Hospital, The University of Tokyo, Tokyo, 113-0033, Japan.
[6]Department of Rehabilitation, Graduate School of Medicine, The University of Tokyo, 113-0033, Japan.
[7]Department of Mathematics, Graduate School of Science and Engineering, Ritsumeikan University, Shiga, 525-8577, Japan.
[8]Division of Integrated Omics, Research Center for Transomics Medicine, Medical Institute of Bioregulation, Kyushu University, Fukuoka, 812-8582, Japan.
[9]Metabolism and Nutrition Research Unit, Institute for Frontier Science Initiative, Kanazawa University, Ishikawa, 920-8640, Japan.
[10]Faculty of Computer and Information Sciences, Hosei University, Tokyo, 184-8584, Japan
[11]CREST, Japan Science and Technology Agency, Tokyo, 113-0033, Japan
†These authors contributed equally to this work.
*Corresponding author: skuroda@bs.s.u-tokyo.ac.jp

This file includes Supplementary Figures S1–S5 and Supplementary Tables S1–S7.

Supplementary Figures

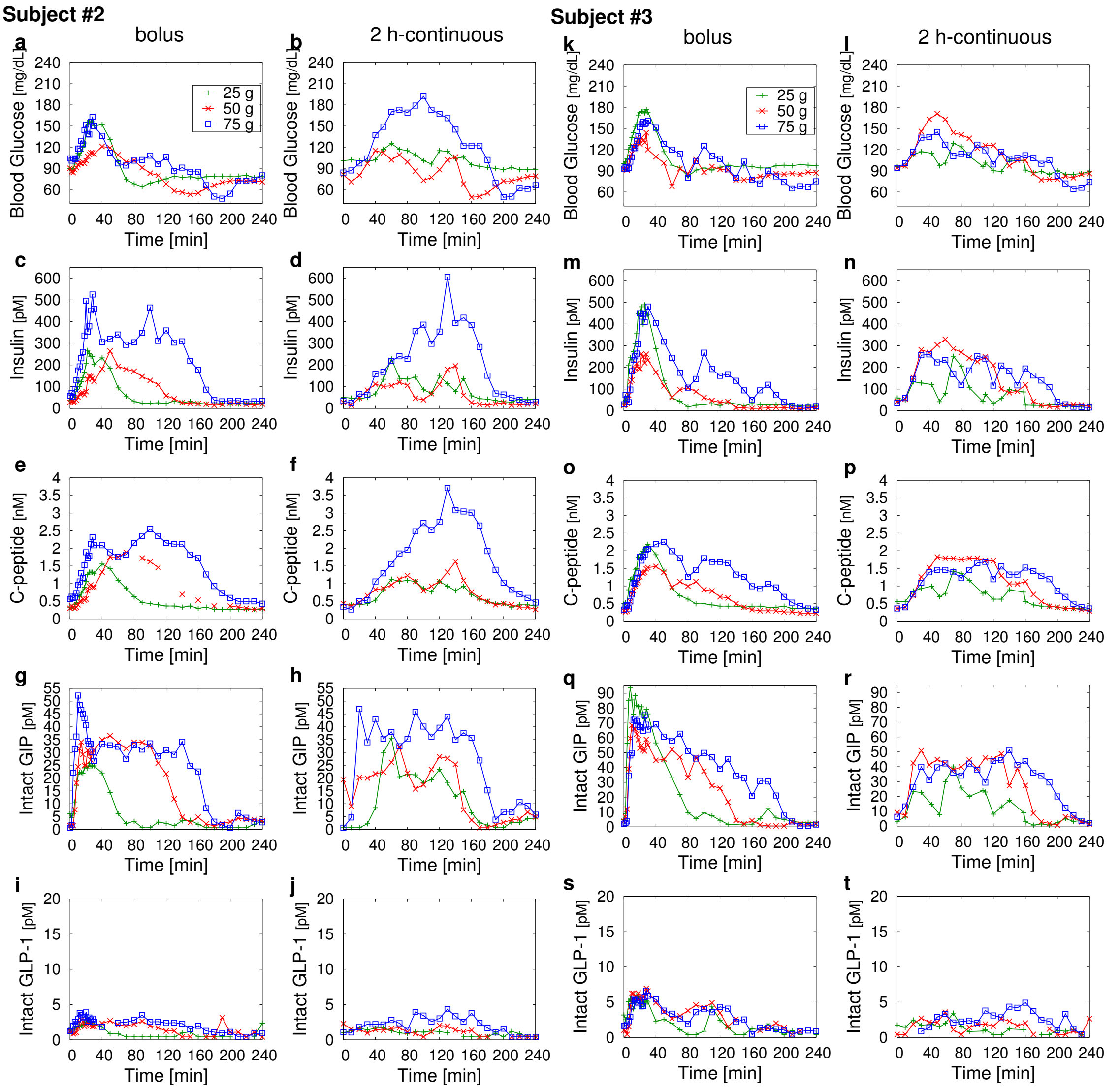

Supplementary Figure S1 Time course data of blood glucose level and blood hormones in subject #2 (**a**–**j**) and #3 (**k**–**t**) by glucose ingestion. **a**, **b**, **k**, **l** Blood glucose. **c**, **d**, **m**, **n** Insulin. **e**, **f**, **o**, **p** C-peptide. **g**, **h**, **q**, **r** Intact GIP. **i**, **j**, **s**, **t** Intact GLP-1. The dose and duration are indicated.

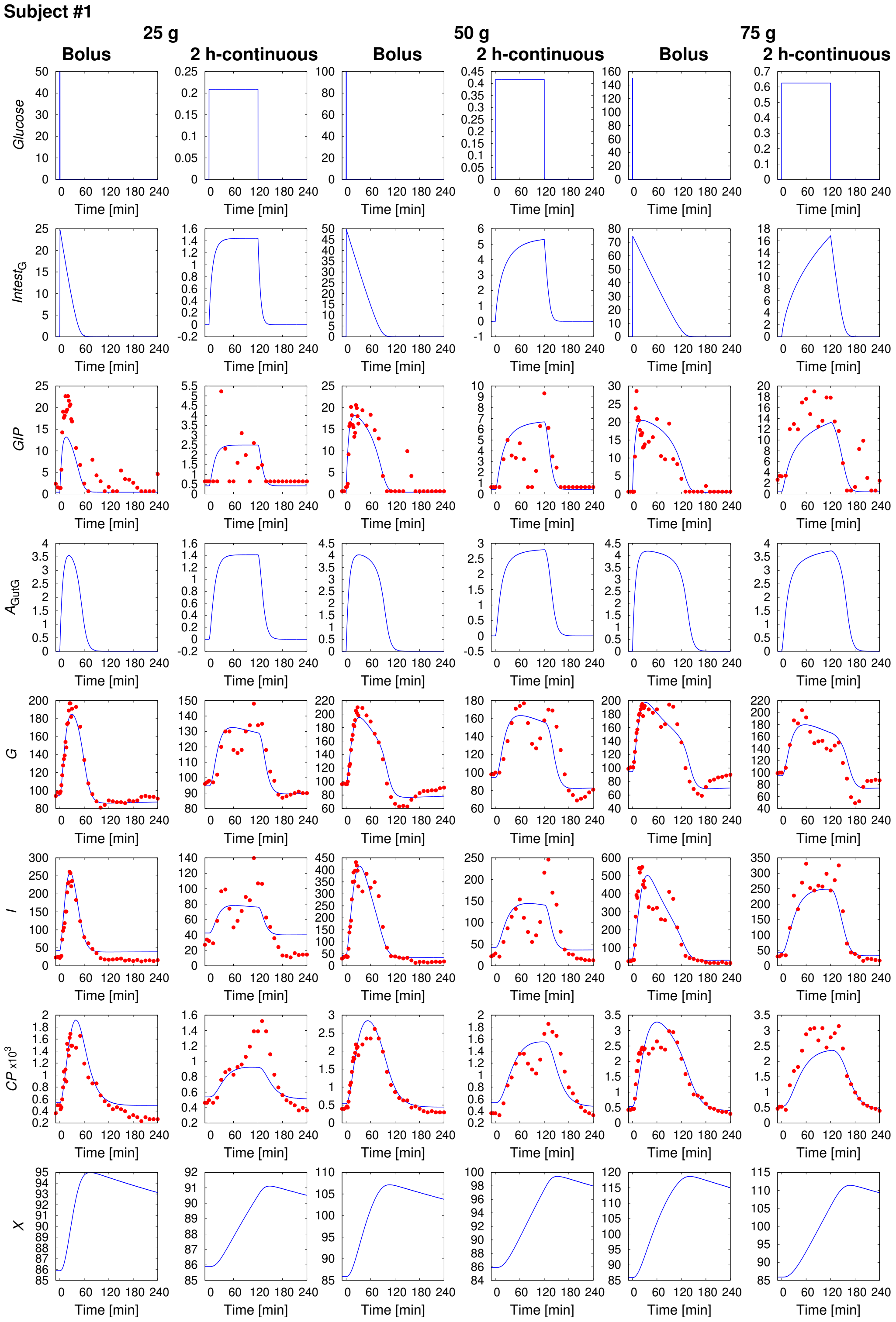
Subject #1
25 g
50 g
75 g
Bolus
2 h-continuous
Glucose
Intest_G
GIP
A_GutG
G
I
CP x10^3
X
Time [min]

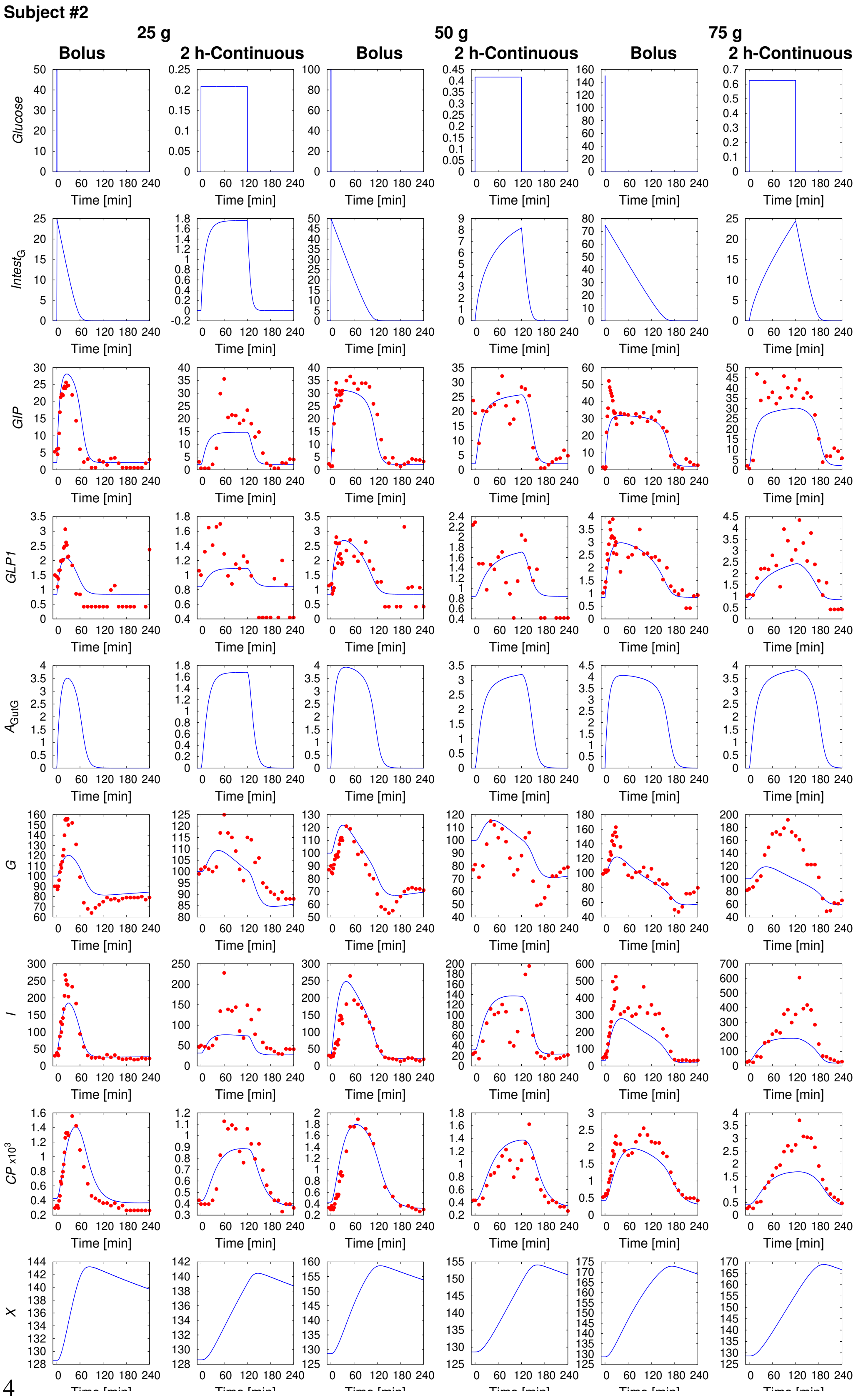
Subject #2
25 g
50 g
75 g
Bolus
2 h-Continuous
Bolus
2 h-Continuous
Bolus
2 h-Continuous
Glucose
Intest_G
GIP
GLP1
A_GutG
G
I
CP x10^3
X
Time [min]

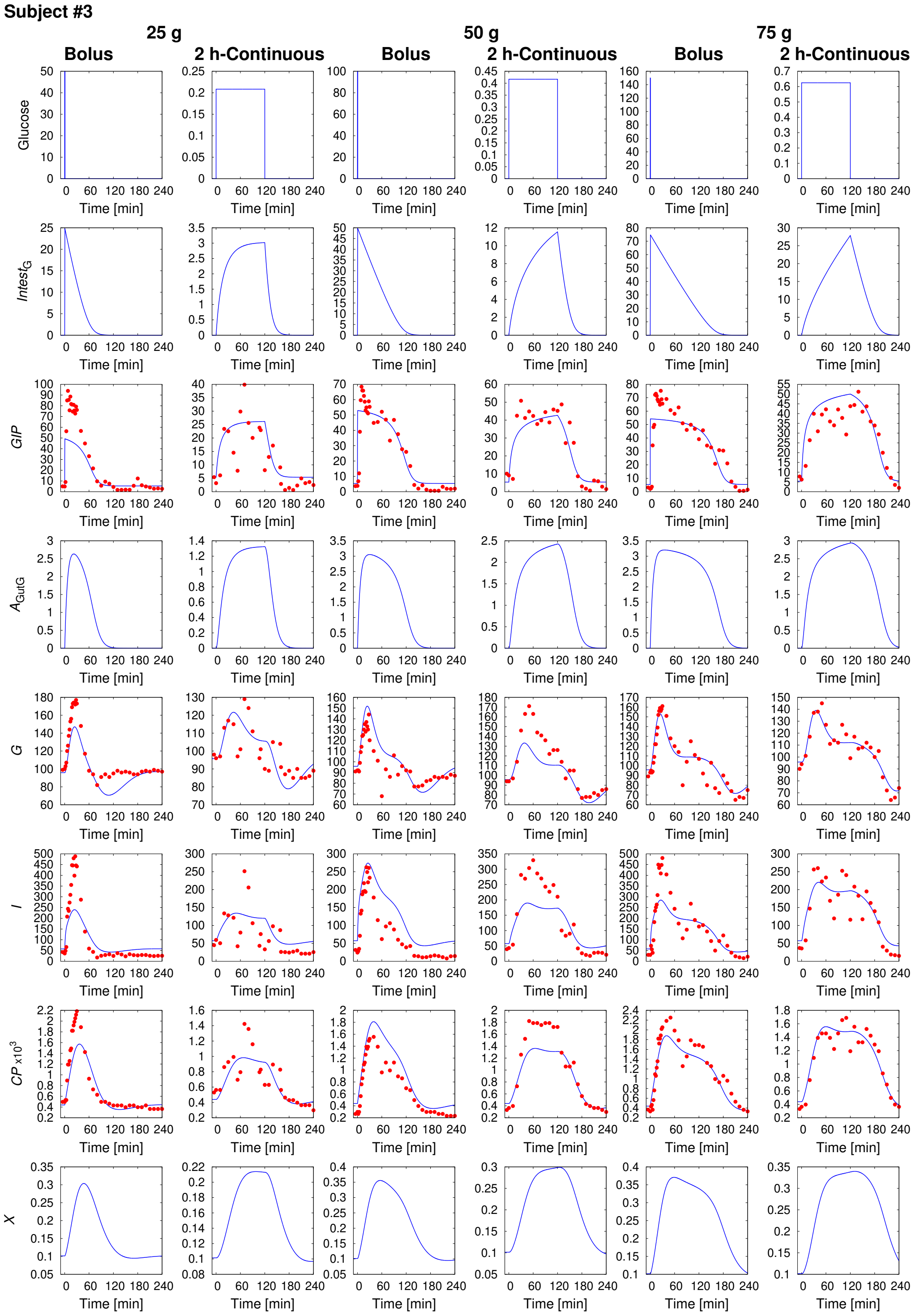

Supplementary Figure S2 Temporal patterns of hormones. The blue lines indicate the temporal patterns of simulations, and the red circles indicate the time course data of experiments. The variable and the ingestion pattern are indicated.

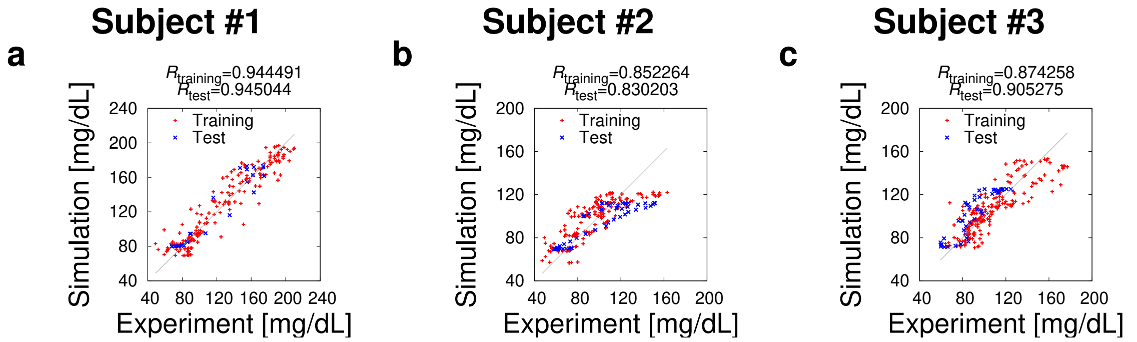

Supplementary Figure S3 Relationship between blood glucose levels in experiment and simulation. **a–c** Scatter plot of blood glucose levels obtained by the experiment and by simulation in subject # 1–3, respectively. “Test” indicates the data set obtained by glucose ingestion according to glucose minimization pattern. “Training” indicates the data sets obtained by ingestion patterns used in the parameter estimations. R_{test} and R_{training} indicate Spearman's correlation coefficient of Test and Training, respectively. We obtained the blood glucose level by blood sampling in (a) and by the flash glucose monitoring system (FGM) in (b, c). because FGM shows the delay of 5 and 20 min, we shifted the time point of the experiment value with the delay that maximizes the cross-correlation function between glucose levels in experiment and simulation (see Methods). R_{training} for each subject is high, indicating that the trained model well reproduces blood glucose level of the experiment. In addition, R_{test} is almost the same as R_{training}, indicating that the model reproduces the data of the validation experiment for each subject.

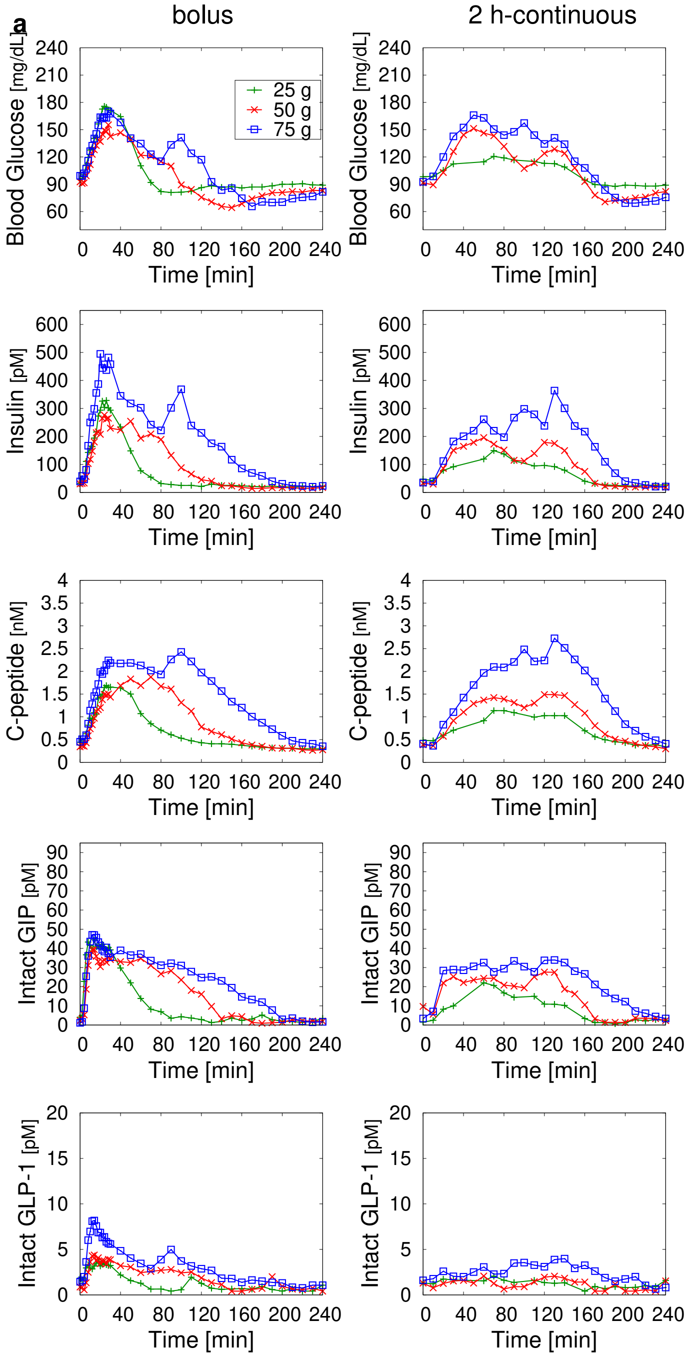
a
bolus
2 h-continuous
25 g
50 g
75 g
Blood Glucose [mg/dL]
Insulin [pM]
C-peptide [nM]
Intact GIP [pM]
Intact GLP-1 [pM]
Time [min]

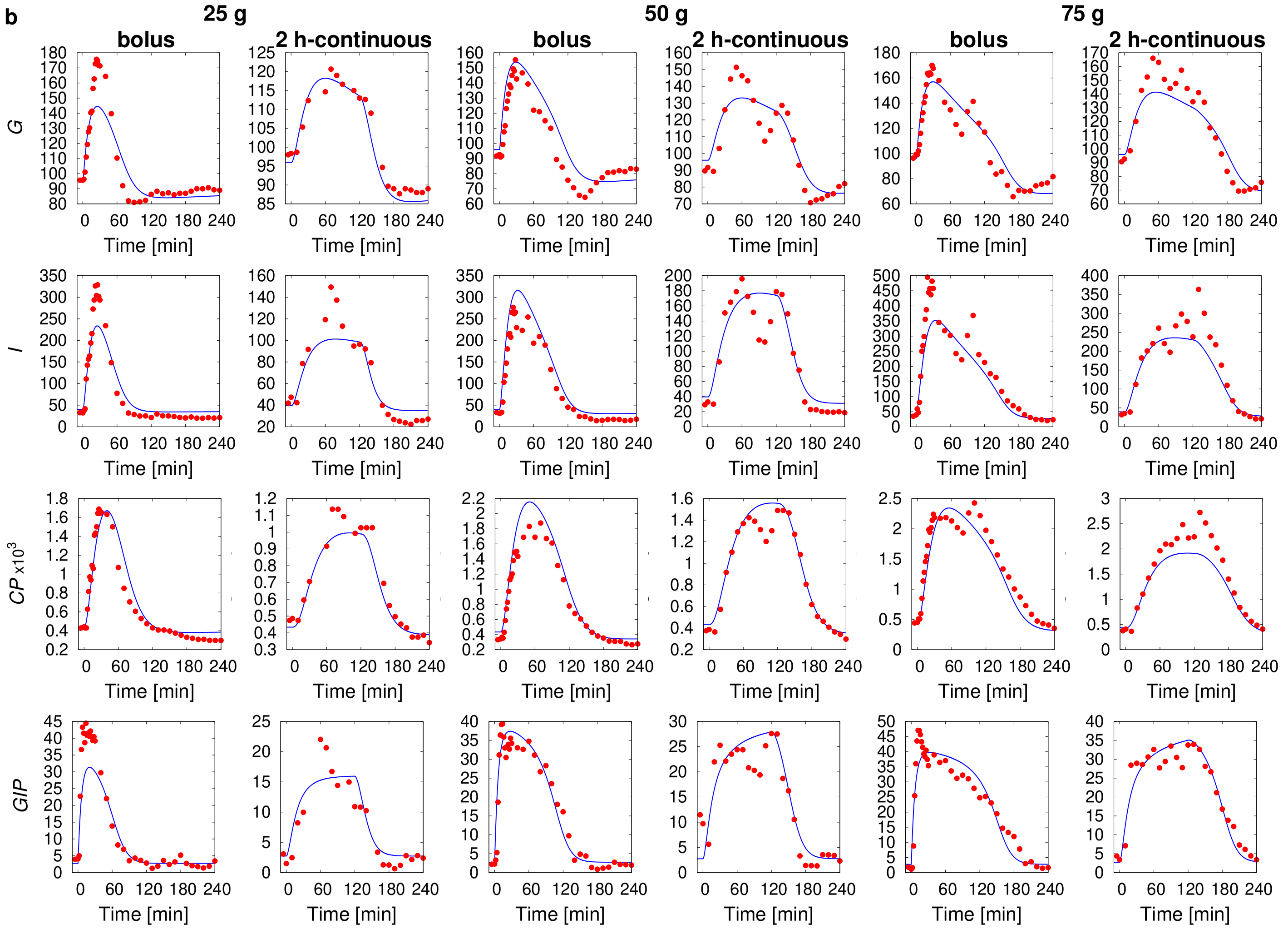
b
25 g
50 g
75 g
bolus
2 h-continuous
G
I
CP x10^3
GIP
Time [min]

c

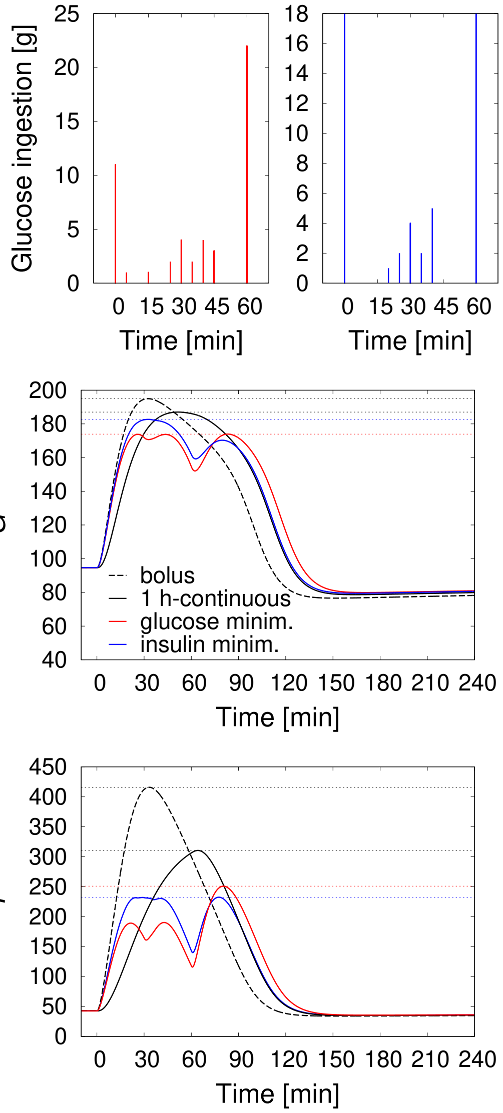

Supplementary Figure S4 The results of subject-averaged model. **a** Time course data of blood glucose level and blood hormones by glucose ingestion, which is plotted in the same manner as Fig. 2. **b** Temporal patterns of hormones, which is plotted in the same manner as Fig. 3b. **c** Optimal patterns minimizing the peak value of blood glucose level or insulin concentration, which is plotted in the same manner as Fig. 4.

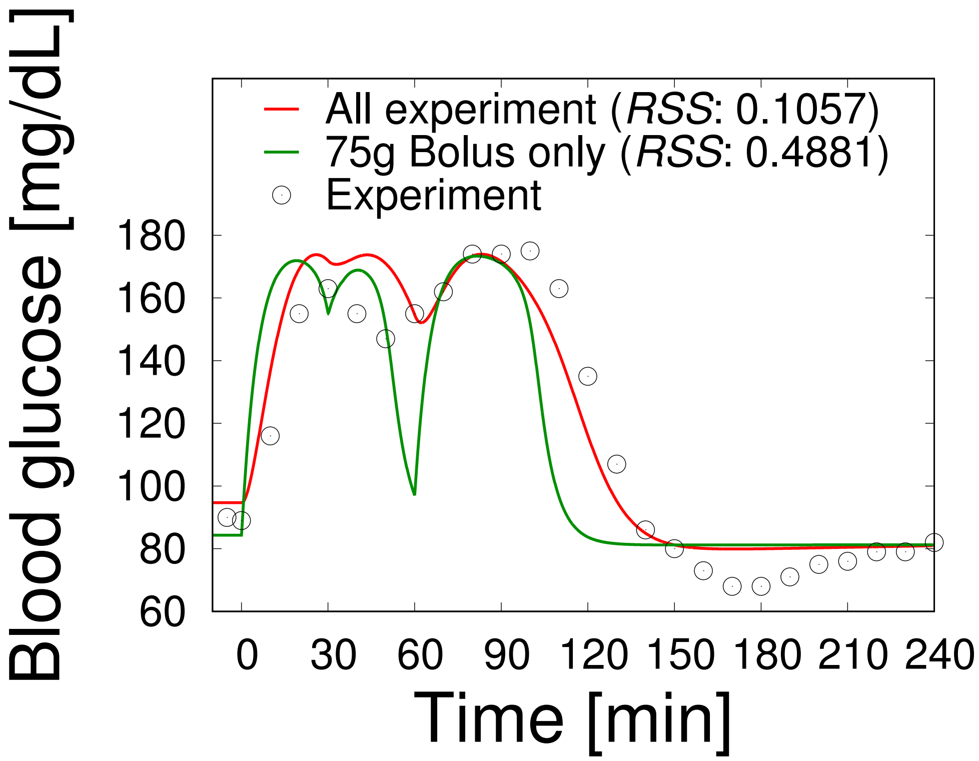

Supplementary Figure S5 Influence of learning data on prediction. Temporal patterns of blood glucose level by simulation with parameters estimated from all experimental time courses data of subject #1, the doses of 75 g, 50 g and 25 g in bolus ingestion and 2 h-continuous ingestion (all experiments, red line) and that from only 75 g bolus ingestion of subject #1 (75 g bolus only, green line) by the ingestion of minimization pattern for subject #1, and time course data of glucose level in experiment for subject #1(black circle). With parameters estimated from 75 g bolus only, RSS (Equation 31 in Methods) of blood glucose level by the ingestion of the minimization pattern was 0.4881, which was larger than that with the parameter estimated from all experiments (0.1057). Thus, the 75 g bolus model was worse prediction accuracy than the model based on multiple doses and durations of ingestion.

Supplementary Tables

Supplementary Table S1 Essential parameters related to insulin secretion and models

Model	a	b	c	d
Full	+	+	+	+
$a = 0$	0	+	+	+
$b = 0$	+	0	+	+
$c = 0$	+	+	0	+
$d = 0$	+	+	+	0
$a = b = 0$	0	0	+	+
$b = c = 0$	+	0	0	+
$c = d = 0$	+	+	0	0
$d = a = 0$	0	+	+	0
$a = c = 0$	0	+	0	+
$b = d = 0$	+	0	+	0
$a = b = c = 0$	0	0	0	+
$b = c = d = 0$	+	0	0	0
$c = d = a = 0$	0	+	0	0
$d = a = b = 0$	0	c	+	0
$a = b = c = d = 0$	0	0	0	0

Parameters a, b, c, and d represent the effects of independent action of GIP, cooperative action of GIP and blood glucose, independent action of GLP-1, cooperative action of GLP-1 and blood glucose, on insulin secretion, respectively (see Methods). Parameters with a positive value (+) means that the corresponding effective action exists for insulin secretion, and parameters with 0 means that the corresponding effective action is negligible and has no impact on the model output.

Supplementary Table S2 AIC and RSS of each model that includes GLP-1 for each subject

	Subject #1		Subject #2		Subject #3		Subject-averaged	
Model	AIC	RSS	AIC	RSS	AIC	RSS	AIC	RSS
Full	3608.175	40.870	2996.241	38.796	3320.154	33.156	2803.865	19.576
$a = 0$	3510.021	36.802	2813.827	31.105	3376.145	35.252	2799.024	19.517
$b = 0$	3588.612	38.072	2825.348	31.531	3322.426	33.306	2960.105	23.166
$c = 0$	3552.763	35.565	2853.344	32.636	3422.711	37.031	2813.971	19.830
$d = 0$	3540.766	35.777	2877.382	33.602	3401.054	36.192	2933.709	22.524
$a = b = 0$	3616.288	35.669	2889.932	35.447	3345.037	34.184	3095.258	26.805
$b = c = 0$	3616.226	38.778	2806.497	30.903	3370.805	35.128	2858.983	20.847
$c = d = 0$	3482.602	32.312	2970.003	37.742	3314.581	33.101	2970.454	23.472
$d = a = 0$	3517.256	40.242	2941.512	36.446	3425.418	37.215	2826.432	20.138
$a = c = 0$	3622.719	33.874	2824.580	31.578	3395.662	34.961	2895.732	21.678
$b = d = 0$	3516.658	33.852	2895.039	34.434	3452.058	37.747	2974.208	23.566
$a = b = c = 0$	3559.894	38.117	2834.068	32.026	3366.047	35.025	2901.403	21.856
$b = c = d = 0$	3549.262	34.220	2815.289	31.310	3338.554	34.022	2963.714	23.354
$c = d = a = 0$	3591.294	38.551	2956.695	37.227	3283.600	32.102	2910.755	22.074
$d = a = b = 0$	3732.652	42.993	2972.021	37.927	3487.038	38.398	2948.805	22.986
$a = b = c = d = 0$	3720.373	39.842	3069.093	42.818	3582.634	41.646	3237.684	31.323

These models include Equation 3 (see Methods).

Supplementary Table S3 AIC and RSS of each model without GLP-1 for each subject

	Subject #1		Subject #3	
Model	AIC	RSS	AIC	RSS
full	2414.135	20.970	2507.173	26.032
$a = 0$	2353.048	19.463	2487.398	25.437
$b = 0$	2385.355	20.273	2524.774	26.705
$a = b = 0$	2613.010	27.092	2706.659	33.930

Models lack Equation 3 (see Methods).

Supplementary Table S4 Initial conditions and parameters for the full model for each subject

	Subject #1	Subject #2	Subject #3	Subject-averaged
$Duod_G(0)$ [g]	0	0	0	0
$GIP(0)$ [pM]	0.4155844	2.1251539	5.3919037	2.7342375
$GLP1(0)$ [pM]	N/A	0.84115319	N/A	1.0157161
$A_{GutG}(0)$ [g]	0	0	0	0
$G(0)$ [mg dL^{-1}]	94.691133	99.957185	95.780054	95.970725
$I(0)$ [pM]	42.588011	31.902810	57.449664	39.550678
$X(0)$	85.895218	128.60289	0.10144112	74.102823
$CP(0)$ [pM]	539.59991	427.70036	438.98192	434.13167
k_2 [pM min^{-1}]	4.5983810	4.3378412	9670.3353	6.5846968
k_3 [min^{-1}]	0.18023886	0.13889005	186.52429	0.15856622
k_4 [pM min^{-1}]	N/A	0.27516783	N/A	77.612643
k_5 [min^{-1}]	N/A	0.096114946	N/A	0.10997268
k_6 [g min^{-1}]	0.65379890	0.53103596	0.54495960	0.63235929
k_7 [min^{-1}]	0.14784588	0.12380265	0.15679182	0.073859059
k_8 [mg dL^{-1} min^{-1}]	2.6942789×10^5	1.0804834×10^3	13.041614	5.1584097×10^3
k_9 [min^{-1}]	1.5073968×10^{-3}	6.5355410×10^{-4}	0.24542875	0.021037536
k_{10} [pM dL mg^{-1} min^{-1}]	0.25940323	0.050933305	0.22902970	0.23701634
k_{11} [pM min^{-1}]	396.27163	190.44481	8.9411148×10^3	414.97404
k_{12} [pM min^{-1}]	0.047876519	0.042988568	0.064978948	0.064938620
k_{13} [min^{-1}]	7.5898373×10^{-4}	7.5071092×10^{-4}	0.031449492	9.1678784×10^{-4}
L_2 [g]	16.242242	2.6176555	4.5132090	6.9492150
L_4 [g]	N/A	18.339666	N/A	7.8353645×10^3
L_6 [g]	3.0801115	2.7280167	4.8946577	6.6393048
L_8	2.1889498×10^4	6.2524076×10^{-3}	5.3676708	3.3737398×10^3
L_{11} [pM]	610.67174	298.54698	1.7950317×10^4	542.62001
GIP_B [pM]	0.41558437	2.1251539	5.3919037	2.7342375
$GLP1_B$ [pM]	N/A	0.84115319	N/A	1.0157161
V [10^2·L]	0.039380487	0.84115319	0.065568801	4.3656032×10^{-3}
a [mg dL^{-1} pM^{-1}]	0	25.136439	0	0
b [pM^{-1}]	0.12450723	0	0.055699537	0.085265750
c [mg dL^{-1} pM^{-1}]	N/A	0	N/A	0.55205096
d [pM^{-1}]	N/A	0.16933903	N/A	4.0266631×10^{-4}

N/A means a parameter is not included in the model (see Methods).

Supplementary Table S5 Glucose minimization pattern and Insulin minimization pattern for each subject

	Subject #1		Subject #2		Subject #3		Subject-averaged	
Time [min]	Glucose minimization pattern	Insulin minimization pattern	Glucose minimization pattern	Insulin minimization pattern	Glucose minimization pattern	Insulin minimization pattern	Glucose minimization pattern	Insulin minimization pattern
0	17	21	7	23	6	14	16	23
5							1	
10						1		
15		1	1		1			
20								
25		3	3		2	1	2	2
30	8	2	1	4	3	3	3	3
35	2	4	3		2	4	2	1
40			1		2	3		
45					4	1		
50								
55								
60	23	19	34	23	30	23	26	21

Amount of glucose ingested at the indicated times is indicated in grams. Each subject ingested a total of 50 g glucose.

Supplementary Table S6 Peak value of blood glucose level for experiment for each subject

	Subject #1		Subject #2		Subject #3		Subject-averaged	
	Simulation	Experiment	Simulation	Experiment	Simulation	Experiment	Simulation	Experiment
Bolus	194.97 [mg dL^{-1}]<br>415.81 [pM]	210 [mg dL^{-1}]<br>431.93 [pM]	121.89 [mg dL^{-1}]<br>248.08 [pM]	162 [mg dL^{-1}]<br>N/A	151.78 [mg dL^{-1}]<br>274.14 [pM]	152 [mg dL^{-1}]<br>N/A	153.83 [mg dL^{-1}]<br>315.87 [pM]	N/A<br>N/A
1 h-Continuous	187.02 [mg dL^{-1}]<br>310.43 [pM]	185 [mg dL^{-1}]<br>216.27 [pM]	120.02 [mg dL^{-1}]<br>212.00 [pM]	168 [mg dL^{-1}]<br>N/A	142.40 [mg dL^{-1}]<br>238.29 [pM]	128 [mg dL^{-1}]<br>N/A	133.10 [mg dL^{-1}]<br>176.9199 [pM]	N/A<br>N/A
Glucose Minimization	173.95 [mg dL^{-1}]<br>250.78 [pM]	175 [mg dL^{-1}]<br>170.71 [pM]	112.36 [mg dL^{-1}]<br>203.52 [pM]	151.67 [mg dL^{-1}]<br>N/A	125.14 [mg dL^{-1}]<br>220.00 [pM]	124.67 [mg dL^{-1}]<br>N/A	137.10 [mg dL^{-1}]<br>234.58 [pM]	N/A<br>N/A
Insulin Minimization	182.68 [mg dL^{-1}]<br>232.33 [pM]	N/A<br>N/A	120.04 [mg dL^{-1}]<br>178.27 [pM]	N/A<br>N/A	140.42 [mg dL^{-1}]<br>199.84 [pM]	N/A<br>N/A	143.12 [mg dL^{-1}]<br>223.54 [pM]	N/A<br>N/A

Top and bottom values in each cell indicate blood glucose level and insulin concentration, respectively.

Supplementary Table S7 Profiles of each subject and hemoglobin A1C (HbA_{1C})

	Subject #1	Subject #2	Subject #3
Age	50s	20s	20s
Sex	Male	Male	Female
Height	168 cm	174 cm	160 cm
Weight	72.8 kg	78 kg	45 kg
HbA_{1C}	5.4%	4.9%	5.4%
